# Neonatal care practices in Buikwe District, Uganda: a qualitative study

**DOI:** 10.1186/s12884-021-03699-4

**Published:** 2021-03-17

**Authors:** Marte Bodil Roed, Ingunn Marie Stadskleiv Engebretsen, Robert Mangeni

**Affiliations:** 1 University of Bergen, P.O. Box 7800, 5020 Bergen, Norway; 2 Makerere University College of Humanities and Social Sciences, P.O Box: 7062, Kampala, Uganda

**Keywords:** Neonatal mortality, Sub-Sahara Africa, Uganda, Neonatal care, Uganda clinical guidelines, Delivery, Breastfeeding, Complicated births

## Abstract

**Background:**

Sub-Saharan Africa is the region with the highest neonatal mortality rate, with Uganda reporting 20 deaths per 1000 live births. The Uganda Clinical Guidelines (UCG) from 2016 have detailed descriptions on care for mothers and their newborns during pregnancy, delivery and the post-partum period. The objective of the study was to identify provider and user perspectives regarding the knowledge of and adherence to the UCG recommendations in aspects of delivery and newborn care, both in cases of normal as well as complicated births.

**Methods:**

The study used qualitative methods with data collection from participant observations, interviews with key-informants and focus group discussions. Malterud’s Systematic Text Condensation (STC) was used for analysis.

**Results:**

The study found low knowledge about the UCG among the health workers. Various discrepancies between performed hands-on-procedures and the UCG were found related to neonatal care practices, including low use of partograms, uncertainty around timing for cord clamping, routine oronasopharyngeal suction of newborns and inadequate implementation of skin-to-skin care.

**Conclusions:**

Continued focus on systemic strategies for further implementation of the UCG is recommended.

**Supplementary Information:**

The online version contains supplementary material available at 10.1186/s12884-021-03699-4.

## Background

Sub-Saharan Africa is the region with the highest neonatal mortality rate, with 27 deaths per 1000 live births, compared to 18 per 1000 globally [1]. These findings give reason for increased attention to the neonatal period which includes newborns from birth and up to 28 days, where the most vulnerable time is the first 24 h after birth [2]. Globally, 15% of women encounter birth complications that can turn into life-threatening situations for the mother or baby or both [3]. Safe pregnancies and deliveries, early initiation of breastfeeding and good quality neonatal care practices and support systems are important interventions to prevent neonatal mortality [4]. Uganda, being a country with high neonatal mortality, is struggling with reducing its neonatal mortality rate of 20 deaths per 1000 live births in 2018 [1].

World Health Organization (WHO) guidelines from 2013 for *Pregnancy, childbirth, postpartum and newborn care* are current recommendations for caring for the mother and her newborn [5]. Based on these guidelines Uganda has developed nationally recognized standard treatments compiled in The Uganda Clinical Guidelines (UCG) from 2016, with derivations also from the Ministry of Health Vertical Programs, and other international guidelines [6]. The guidelines included are for mothers and their newborns during pregnancy, delivery and the post-partum period, both in cases of normal as well as complicated births. The use of partogram is recommended for monitoring of safe deliveries and early recognition of birth complications. For the postnatal period the guidelines include practices on suction, delayed cord-clamping (DCC), skin-to-skin care for the newborn, also called kangaroo mother care, early initiation of breastfeeding and other recommended and unrecommended procedures in the mother and the child [5, 6]. Previous studies from Uganda show low adaptation and use of partograms with findings revealing 68% incomplete plotting of date and time for membrane rupture [7]. Studies on timing for cord clamping in Uganda show promising results with the majority of findings revealing clamping of the cord between 1 and 3 min, with medians of 50 and 76 s after birth, which is superior to results found in other low-resource countries [8, 9]. Practicing of skin-to-skin contact between mother and baby has been found non-existent or minimal [10], whereas early initiation of breastfeeding in Uganda is estimated to 66% [11]. Adherence to recommended guidelines are important measures in reducing the neonatal mortality rate, and continuous research is needed to diminish the know-do gap in maternal and newborn health care. Uganda was included in the first group of nine countries to be involved in The Quality of Care Network established in 2017, which mission was to look at the quality of care in maternal, newborn and child health, how it is received by patients and how it is delivered by health workers. The network has provided an implementation guidance with strategies for improvements and sustainability of quality care at all levels of the health care system, in which continuous measurements of quality is one of them [12]. Earlier research show poor policy implementation of newborn care practices in Uganda [13]. In order to improve conditions for *all* mothers it is necessary to understand how the health system works in rural Uganda. This study aimed at identifying provider and user perspectives regarding the knowledge of and adherence to the UCG recommendations in aspects of delivery and newborn care. Facilitators and barriers for mitigation of the know-do gap related to UCG were explored both in cases with normal and complicated delivery and post-partum histories in Buikwe District, Uganda.

## Methods

### Design

The study used a qualitative approach with data collection through triangulation from the most commonly used qualitative methods of participant observations, interviews with key-informants and focus group discussions. A triangular qualitative approach was chosen in an effort to get a nuanced and deeper understanding of the topic [14].

### Setting

The study was conducted in 7 villages of Buikwe district, including the urban centre of Nyenga and six of its surrounding 10 sub-villages. The six sub-villages were selected after conclusion of a monthly village committee meeting in Nyenga town, where the leaders of the sub-villages present were approached for information about the study and where appointments were made to meet with the leaders in their home villages for further planning of the research. The local leaders were given a general information letter of the purpose of the study and asked to help with identification of mothers with newborns in the villages, in addition to logistics and provision of location for the focus group discussions. Saint Francis Hospital and Saint Francis School of Nursing and Midwifery are connected to each other within the same location of Nyenga town. The students at the school are provided with full boarding and have rotating internships within the various wards of the hospital. Kabizzi village includes one of several health centres for the public in the area, providing both in-and out-patient services with a two bedded maternity room [15, 16]. In cases of referrals most patients go to Saint Francis Hospital in Nyenga, or other private or public hospitals upon request from the patients.

### Population

**Fig. 1:**
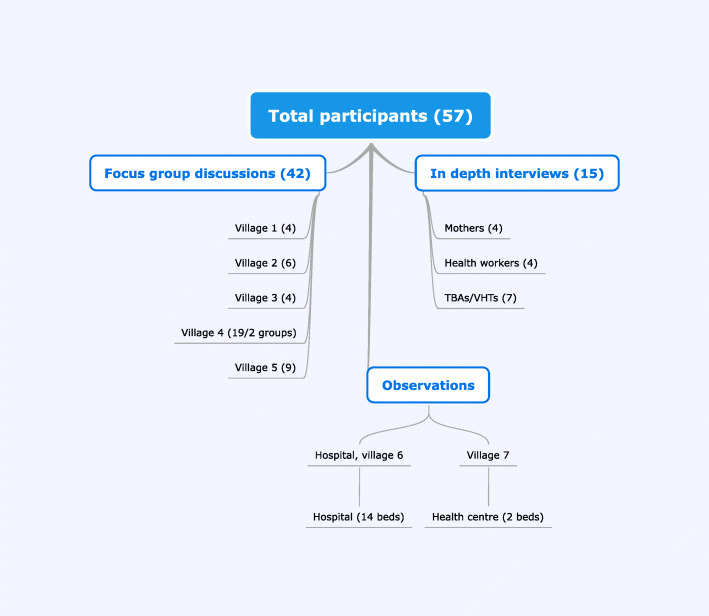
Flow chart explaining data collection and distribution of participants

Selection of participants was done through purposeful recruitment from St. Francis Hospital, Kabizzi Health Centre and the defined villages. The Hospital and Health Centre reported an average of 500 and 30 births each year, respectively. Both facilities had one midwife on duty, and the hospital also had an assistant midwife and generally 3–4 nursing-and midwife students on rotating shifts in the mornings and afternoons Fig. 1.

### Sample

The total number of participants was 57. The key-informants amounted to 15, and 42 mothers attended focus-group discussions. Except for the health workers, all participants were subsistence farmers, with some having small additional businesses. The number of patients during observations was not counted (Table 1).

**Table 1:** Background information of participants

Subjects	Data source	Numbers	Age	Education
Mothers	Interviews	4	15 to 45	Primary to secondary
TBAs		4	50 to 80	None to primary
VHTs		3	50 to 80	Primary
Health workers		4	19 to 35	Certificate level
Mothers	Focus group discussions	42	16 to 45	None to secondary
Hospital/Health Centre. (Health workers, students, mothers)	Observations	Unknown	15 to 80	Primary to PhD

### Data collection procedure

Two local research assistants, male and female, fluent in both English and Luganda with bachelor’s degrees in social sciences were recruited for logistic organization, moderator roles in focus group discussions, recruitment of key-informants and transcriptions and translations of recordings in local languages. The researchers were trained and educated in the research topic and interviewing techniques beforehand by the main researcher.

Key-informants were chosen purposefully because of their involvement and every-day encounter with deliveries and neonatal issues. Mothers were purposefully selected on the inclusion criteria that they had given birth within the past month.

In-depth interviews were done with mothers and other key-informants such as students in nursing and midwifery, midwifes, traditional birth attendants (TBAs) and village health team workers (VHTs) (additional file 1). TBA’s and VHT’s are the key frontline service providers in rural areas of Uganda, often being the first, and sometimes the only contact point for mothers in need of help and guidance on maternal issues [17, 18].

The time period for data collection was from January 15th to February 25th, 2019. Qualitative semi-structured interviews with key-informants in English (*n* = 5) were conducted face-to-face by the principal investigator or if in other languages by one of the two research assistants (*n* = 10). The interviews with health care workers took place in the hospital or health centre. Three interviews with mothers took place in the hospital, whereas the other interviews with mothers, TBA’s and VHT’s took place in their private homes or outdoors within the various villages. For most interviews the primary investigator was present for observations and note taking. The duration of the interviews ranged from 10 to 30 min.

All focus group discussions were conducted by one of the research assistants in Luganda in an outdoor location provided by the local leaders in the selected villages. The focus group discussions lasted from 40 to 60 min. The principal investigator, who is a nurse, was present for participant observation in the maternity ward at the Hospital and Health Centre for 6 weeks. During this period she spent the mornings in the Hospital, taking rounds with the doctor on duty and observing deliveries and postnatal care and procedures. The afternoons were spent in the Health Centre, observing deliveries and aiding the staff when necessary. Notes were written continuously.

Audio-recorded interviews were transcribed word by word into Microsoft Word documents within 2–4 days. Interviews and focus group discussions conducted in Luganda were transcribed first in Luganda, then translated into English by the same research assistant. Proof readings, consisting of listening to the recordings in Luganda while reading the Luganda and English transcriptions, were done by another colleague fluent in both English and Luganda. No discrepancies were found in the translations. Notes from observations were typed into Word documents upon completion of the observation period.

### Instruments

Locally pre-tested semi-structured interview guides were used for interviews with key-informants and for focus group discussions (additional files 1 and 2). The UCG were used as background for the inquiries and structure from delivery through the neonatal period. The UCG guidelines involve hourly monitoring of the mother’s vital measurements, including the use of partograms and curves [2, 19]. Detailed description of newborn procedures such as delayed cord clamping, suction, kangaroo care and early initiation of breastfeeding are included, thus our interview guide focused on these stages connected to birth: Monitoring of labour using partograms gives a better indication of labour progress for easy recognition of early complications [19, 20]. Delayed cord clamping (DCC) refers to clamping later than 60 s after birth or when cord pulsation has ceased, contrary to early cord clamping (ECC) which take place within 1 minute after birth [21, 22]. Suction of newborns is a method used to clear secretions from the oropharynx and nasopharynx through the application of negative pressure via a suction catheter or bulb syringe, and is not recommended for uncomplicated births [23]. Skin-to-skin care, also called kangaroo mother care, is when the newborn is placed on the mother’s bare abdomen or chest and should last for at least 1 hour or until after the first breastfeed [24]. Early initiation of breastfeeding meaning initiation within the first hour after birth. The UCG has implemented training and dissemination in the current UCG procedures regarding these issues, which when followed have been documented to reduce the risk of neonatal deaths and are therefore some of the most important focus areas for lowering the death rates of newborns [25, 26].

### Data analysis

The analysis was performed using the inductive method developed by Malterud called Systematic Text Condensation (STC), modified from Giorgi’s psychological phenomenological analysis. The method of STC appealed to the researcher with its’ inductive approach where the themes from the findings reveal themselves to the researcher following a step-by-step process which involves reflections and alternations before reaching the final presentation [27]. The transcribed text and observation notes were read in-depth several times to find emerging themes that revealed themselves to the researcher during the reading process. The documents were copied into the NVIVO 12 pro software program for better structuring of the analysis and the emerging themes were sorted into code-groups. Further decontextualization consisted in extracting another level of sub-code groups from the code-groups, to create a systematic overview including facilitators and barriers for adherence to the UCG. These structures were used as bases for condensed narratives of the findings, with a “*golden quote*” to validate the narrative. In this study the principal investigator was a female nurse (MBRL) from Norway who had previously lived short term in Uganda. She was doing the primary data collection, coding and analysis of the data. IMSE was co-reading the raw-data and discussed the main themes with the first author. RM contributed with co-development of the interview guides, acquisition of data and discussions of main themes with the first author.

After analysis, the respondents were invited for a dissemination meeting followed by a traditional celebration. The researchers disseminated the key findings and their interpretation of the data and asked the participants openly about any needs for modification of their understanding. The participants were invited to comment, modify and ask questions about the findings. The participants did not know the status of the other participants as informants and all informants were informed about this planned dissemination meeting at inclusion.

### Ethical considerations

Participation in the study was fully voluntary. Each participant in the focus group discussions was given a number for recognition and was asked not to share private information gained during the sessions outside the group. Before the interviews and focus group discussions participants were offered snacks and refreshments. They were also reimbursed for transportation costs up to 15,000 UGX (equals to 4 USD). No extra money was given for participation, but the mothers attending focus group discussions received a piece of locally made baby clothing after the session.

## Results

The following section will display results extracted from the final code group labelled *Pregnancy and Birth,* which emerged during the analysis using Malterud’s Systematic Text Condensation where facilitators and barriers in adherence to guidelines were focalized [27]. Based on the predominate responses from participants and main observation findings, the study found discrepancies between the UCG and performed hands-on-procedures related to neonatal care practices. Key findings disclosed infrequent and rare usage of partograms for monitoring of deliveries, deviant or uncertain timing for cord clamping and routine suction of newborns performed against recommendations by professional health workers, whereas TBAs operated in consistency with UCG. On the use of skin-to-skin care, the routines varied, and although the practice was customarily initiated, it seldom lasted for the recommended time span. Initiation of breastfeeding within the first hour after birth was routine, unless complications occurred, in which cases the mother and baby could be found separated from each other for up to 3 days. The results are thematically presented in the table below (Table 2).

**Table 2:** Overview of results from various respondents’ perspectives

	Parto-gram use	Timing for cord clamping	Suction/wiping of newborns	Skin-to-skin care	Initiation of breastfeeding	Complications
Mothers	N/A	Uncertain	Not mentioned	Various experiences	Mostly as recommended	Deviant from UCG
Health workers	Rarely used	Deviant from UCG	Against UCG recommendations	Limited skin-to-skin contact	As recommended	Marginal resources and infra-structure
TBAs/VHTs	N/A	Various procedures	As recommended	Uncertain	As recommended	Hospital referrals

None of the respondents confirmed knowledge about UCG, but the researcher found the printed guidelines present in both the Health Centre and the Hospital. When asked to describe what happens during labour and after birth, the respondents talked of procedures related to vital examinations of the mother, baby’s body warmth, hygiene, cord clamping, resuscitation and necessary injections and medicines. These procedures were also closely observed by the researcher when present during and after deliveries.

### Monitoring of delivery

#### Health workers perspective

In-depth interview with health workers about monitoring of women in labour showed knowledge about vital observations. They would check the heartbeat, is it present? Is the baby still alive? They would listen to the heartbeat of the baby through the fetoscope: Is the heart beating weak or faint? Or is it maybe beating too fast? They explained about taking the fundal height and procedures for vaginal examination:

“*Did she take the circumcision, you see some people have, is she having a normal vulva? So, I do the cleaning, after that I go inside, I must check - is the vagina hot? If it is hot it is also an alarming thing. So, it has to just be warm and moist. Because if she’s in labour and the vagina is actually dry, it is also a problem*”. (Midwife 25–35 years).

Observations in the Hospital and Health Centre found common use of freehand journal writing as documentation of delivery. Although charts and partograms were available, they were rarely or unsystematically used which could sometimes cause difficulties or delays when searching for specific vital data or monitoring of a patient’s delivery progression.

#### TBAs’ and VHTs’ perspective

Both VHTs and TBAs assisted women who came to ask for help in labour. They would check the mother for signs of diseases and had low thresholds for referrals to hospitals if the mothers were showing signs of complications.

“*A woman when she produces, and you check her that she’s ok, you get a flask of warm water and mix it with tea leaves because it helps her to produce well. When a mother is in labor pain and when there is no one to help her, I do give a hand to her to help her deliver well*” (VHT 50–80 years, village 1).

### Cord clamping – timing

#### Mothers’ perspective

A number of the mothers told stories of how they were unable to reach qualified help when the time for delivery was near. Reason could be transport cost, unsafe environments, darkness, or long distance. Nevertheless, home births could have the advantage of facilitating delayed cord clamping:

*“I produced outside the house, so they took the baby inside. When I was done with giving birth, I knelt down for the remaining’s of the placenta to come out. They got the baby to cut the cord, after they covered the baby and they took the baby inside*”. (Mother 25–35 years, village 3).

#### Health workers’ perspective

Optimally, from the health workers’ perspective, clamping of the umbilical cord was done using forceps, but from observations done by the researcher, the cut-off ends of surgical gloves were frequently used for cord clamping in cases where the forceps had not been sterilized or were unavailable. The clamping and cutting of the baby’s umbilical cord was normally performed on the mother’s abdomen *immediately* after birth. Further shortening of the cord was then done in the infant warmer, or sometimes also on the abdomen of the mother:

“*The baby is placed on the stomach of the mother, then you get two forceps, then you use them to attach to the umbilical cord, then you cut*”*.* (Nursing student 19–21 years).

The “warmer” is an electrically heated machine where the newborn was placed after birth while the health workers cleaned the mother and where procedures on the baby were performed, thus creating a barrier for skin-to-skin contact.

#### TBAs’ perspective

Traditional birth attendants naturally did not have the equipment and resources available at the health facilities, but several of them had attended training with medical doctors and had been taught what steps and measurements to take after attending to a delivery. The procedure of cord-clamping varied from village to village as described by two different attendants below:

*“I shorten the cord before the remaining of the placenta comes out because that’s how the doctors told us”*. (TBA 50–80 years, village 1).

*“I help the mother to remove the remains of a placenta from the mother’s womb then after removing it I cut it off”.* (TBA 50–80 years, village 5).

### Oronasopharyngeal suction

#### Health workers’ perspective

Routine suction of the nose and mouth was performed various places. Sometimes it happened on the abdomen or chest of the mother, whereas other times it could be performed on a separate bed or in the infant warmer. Students interviewed also confirmed being taught how to routinely suction newborns.

“*After clamping, if the child is OK, you resuscitate, you first suck out the secretion from the noise and the mouth to open the air to breath, you have to resuscitate with the barb syringe, and you remove the mucus here in the mouth. If you don’t, that’s when you find some babies having fever, then flu at an early age*”. (Midwife 25–35 years).

#### TBAs’ perspective

As a variation to oronasopharyngeal suction by bulb syringes mentioned by the health workers, another method used by the TBAs, facilitating guideline recommendation, was described as follows:

“*If the baby has been born, immediately you clean the baby very well with a clean cloth because they (health workers) provided us with them, so I use those clean clothes to clean the baby in the ears and mouth for the baby to breath well”*. (TBA 50–80 years, village 1).

### Skin-to-skin care

Placing the newborn baby on the mother’s abdomen or in her arms immediately after birth is often referred to as kangaroo care, skin-to-skin contact or *kulubutu* in Luganda.

#### Mothers’ perspective

Many mothers confirmed having the baby put on their chest after birth and they spoke of the importance related to hygiene and warmth. Others had experiences of being separated from their newborns for unknown reasons.

“*after birth they cut the cord, they covered the baby and placed the baby on the bed”.* (Mother 15–25, village 3).

#### Health workers’ perspective

From observations, the newborn was routinely put on the mother’s abdomen immediately after being born, but there were often only a few minutes of contact before the baby was moved to the infant warmer for further procedures, then wrapped in several layers of blankets before returned to the mother.

*“After shorten the cord you wrap the baby … .remember you do all that (procedures) when you put the baby in the warmer. At least you maintain the temperature that was in the uterus. Then you wrap the baby in warm clothes and give it to the mother for breastfeeding.”* (Midwife student 19–21 years).

In the Health Centre, where there was no warmer, the baby was more likely to be placed in the arms of the mother during the cleaning process or given to a family member or attendant for safekeeping.

“*when the baby is born you have to get a cloth and put on the abdomen of the mother, as we did it yesterday … and then the baby has to cry, and you place it on the abdomen”.* (Midwife 25–35 years).

#### TBAs’ perspective

When someone gives birth from home with the help of a TBA, the option of putting the baby somewhere other than on the mother’s body was limited. Even so, the practices varied:

“*… you clean up the baby and place the baby somewhere and then clean up the mother”.* (TBA 50–80 years, village 2).

### Initiation of breastfeeding

Overall knowledge about the usefulness of breastfeeding was found among all the participants in the study, and the option of not breastfeeding seemed none-existent, or even unheard of. Time of initiation was found in accordance with the UCG in most of the observed cases and no adverse knowledge or recommendation were detected on this subject. Mothers who had faced complications during or after birth, were naturally more likely to initiate breastfeeding later than the recommendations.

### Complicated births

#### Mothers’ perspective

Some mothers had experienced complications that lead to emergency Caesarean section surgeries, and this could be a challenge. They lost control of the situation and the initiation of breastfeeding was delayed. Sometimes it was the baby who faced complications, and a mother of three who delivered in a hospital explained how she was separated from her baby for three days before the initial breastfeeding occurred:

*“I remained in the ward for the mothers. This one they took him in the room. Special care room, for these children, and they worked on him for three days before I saw him, yeah ( …*) *for three days, after there, they give me to breastfeed, to start breastfeeding him”.* (Mother 25–35 years, village 7).

#### TBAs’ perspective

The traditional birth attendants interviewed were aware of the risks connected with childbirth, and when asked about how they dealt with complications, most of them said that they refer the mothers to the hospital.

*“… When you first bleed with blood when about to give birth that’s a bad sign and it is not good, when I notice that, immediately I refer them to the hospital, when still in labor pain I am the one to escort them to the hospital …”* . (TBA 50–80 years, village 5).

One traditional birth attendant said she used her herbal medicine to help with complications and one respondent described how she dealt with retained placenta:

“*When the placenta refuses to come out quickly, I have to place the baby on the mother’s breast when I have cleaned it well, and when the baby sucks the breast it helps the placenta to come out quickly*”. (TBA 50–80 years, village 5).

During the dissemination meeting which took place in November the same year, the participants’ feedback about the study was unanimously positive. Many expressed gratitude for receiving information about updated research and guidelines and gave constructive feedback to the researchers with suggestions for improving quality of maternal health care in Uganda. One recommendation suggested was structured interchanging of knowledge and practices between TBAs and midwives. Another suggestion was to focus more on breastfeeding routines and -problems during antenatal classes.

## Discussion

This study aimed at identifying provider and user perspectives regarding the knowledge of and adherence to the UCG recommendations in aspects of delivery and newborn care, both in cases of home births and deliveries within health facilities. Barriers and facilitators to adherence with the UCG were explored both in cases with normal and complicated delivery and post-partum histories in Buikwe District, Uganda.

Results from the study show that the UCG were unfamiliar to the health workers in the study, although responses and actions from the health workers were found to be in accordance with the guidelines in many areas [6]. Other studies have shown low adherence to guidelines regardless of knowledge thereof [28]. Areas for improvements involve better documentation of labour progress and higher compliance with recent research-based updated guidelines on specific newborn procedures such as timing of cord clamping, suction and skin-to-skin care as described in the UCG.

The observed minimal documentation on charts and partograms during deliveries could be attributed to time restrictions and health workers’ confidence in own clinical observations. However, assessment of labour progress is important, and especially so if labour is prolonged or if complications occur. Circumcisions of women is illegal in Uganda and on the decline, but some tribes and individuals are still performing this procedure which is a contributor to birth complications [29, 30]. Monitoring of labour using partograms gives a better indication of labour progress, and together with knowledgeable and observant midwives it may prevent unnecessary emergency Caesarean sections [19, 20]. A Ugandan study from 2009 on the use of partograms found poor use and lack of training among the staff on how to use them [19]. Similar results were found in a recent study from 2019 [7], communicating the need for continued attention to and improved measures for implementation of this important monitoring tool. Support supervision in health facilities have shown positive results in quality improvements on maternal and newborn health services and practices [31].

Clamping of the umbilical cord in the Hospital and Health Centre was performed immediately after birth and normally before the presentation of the placenta. These findings diverge slightly from recent Ugandan studies that found timing of cord clamping satisfactory, although with differentiation in the results to the positioning of the baby, whether it was placed above or below the level of placenta, hence allowing the gravity to maximize the benefits of postnatal pulsations in the cord [8, 9]. Results from this study reveal that some of the traditional birth attendants waited for the placenta to come out and thereafter cut the cord. Benefits from delayed cord clamping has been found both for pre-term babies as well as for those born at term. Qian et al (2019) found benefits involving higher Hemoglobin levels and reduced anemia. Also, higher levels of iron supplies have been found in infants up to 6 months [32]. Another study from 2019 found higher levels of Oxygen in babies with delayed cord clamping than in those who had early cord clamping, in addition to slower heart beat and higher Apgar scores [22]. Studies have not found an elevated risk of postpartum haemorrhage in the mothers, and the benefits to the baby is equally high or higher when delivered by Caesarean section [21, 22, 32, 33].

*The procedure of oronasopharyngeal* suction was not in alignment with the Uganda Clinical Guidelines which states that it is part of “*Harmful and ineffective resuscitation practices”* (UCG 16.5.1) [6]. The routine suction performed by the health workers, was believed necessary to allowing the baby to breathe. The reasons behind the suction practices could be related to the curriculum of the schools, or just a continuous practice from earlier recommendations. The practice of routine suction of newborns has been found to cause side effects like bradycardia and apnoea, although not with the use of a bulb syringe [34]. Wiping the baby’s nose and mouth with a clean cloth after birth, as more often performed by traditional birth attendants, is in accordance with recent studies from the U.S. and Austria, where wiping was found superior to suction, unless the baby had difficulty breathing [35, 36]. Hence, recommendations on cooperation between professional health workers and TBAs should not be underestimated [9]. Issues with proper cleaning and reusage of suction bulbs may be an additional unnecessary trigger of newborn infections [37].

The use of skin-to-skin contact after birth was common practice, although the time period for the skin-to-skin connection could be only a few minutes. The UCG have divergent approaches to skin-to-skin care. In the description of the “3^rd^ stage of birth” it recommends “*to wrap the baby in warm towels and give to the mother to introduce breast feeding*” whereas in the section for “Care of Baby Immediately After Delivery”, the recommendation is to “*keep baby warm with skin-to-skin contact*” [6]. UNICEF recommends skin-to-skin contact for 1 hour, or until after the first breastfeed [24]. The routine of placing babies in a warmer for the following newborn procedures seemed to create a barrier for further skin-to skin contact. One explanation for this practice may be due to the marginal bedding on the delivery beds. The mother is required to bring a plastic sheet to cover the delivery bench, which creates blood and other substances to stick to the body of the mother and require thorough cleaning before she can be moved to another bed. The use of one-time absorption sheets for delivery surfaces would seem more beneficial and shorten the time span of separation between mother and child, but this may be an economical issue [37]. Low-cost interventions of training health workers in the importance of skin-to-skin contact has been successful in other parts of Uganda and should be considered [10].

Sometimes separation between the mother and the newborn is inevitable when complications occur, whether during or after delivery. Ideally the separation period should be as short as possible [38]. Separation between the mother and the newborn may create insecurity and unnecessary worrying for the mother and delay the bonding and breastfeeding initiation. Studies have also revealed separation as a cause for stressors in the newborn which could lead to neurodevelopmental problems [39–41]. The study found cases of separation from the newborn for up to 3 days after birth complications. There may be unknown medical reasons for the separation in this study, but further inquiries are recommended to ensure this is not standard procedure. Findings from other studies show common use of kangaroo-mother care when mothers reside in the same ward/hospital, but not always incorporated if baby stays alone [37].

### Study strengths and limitations

Limitations of the study include possible bias as the principal investigator was a foreign person which may give participants incentives to over- or under exaggerate their answers. However, being a foreigner is sometimes perceived as non-threatening by the interviewees who may speak more freely than with somebody belonging to the same health system that may criticize their practices. There was no initial research done to investigate whether the UCG had been attempted implemented in the chosen health facilities. Background knowledge of this fact may have altered questions in the interview guide, conversely, it could have made the researcher more biased to the health workers’ answers. There were few midwives included in the study because of the low number of midwives on the chosen study sites. This may have influenced the results from the health workers’ perspective as responses from students with less experience were overrepresented. Credibility of the study was sought obtained by using local translators and trained interviewers of both sexes, in addition to proof readings of transcriptions. Findings from one focus group discussion and one interview were typed from notes only, due to issues with electric power and technical difficulties. This may have caused loss of information otherwise recorded. Corresponding results from observations and interviews gave stronger dependability of the study, although no observations were done for home births. Based on previous research from rural settings in Uganda the results from the study are considered transferable to settings with similar context and clienteles.

## Conclusion

Findings from the study show the importance of continued awareness and focus on systemic strategies for implementation of the UCG recommendations. Implementation of the UCG in the curriculum of nursing-and midwife education is advisable. Low-cost interventions such as various teaching methods targeting promotion in practical use of the UCG are recommended as measures for mitigation of the know-do gap in neonatal care practices. Diverging practices between health workers and TBAs, give indications for closer coalitions between the two branches in maternal health care. Further research on mother-infant contact after birth complications is recommended.

## Supplementary Information

**Additional file 1.** Interview guide for key-informants

**Additional file 2.** Interview guide for focus group discussions and mothers

## Data Availability

Questions regarding the datasets can be discussed with the corresponding author on reasonable request.

## Abbreviations

DCC: Delayed cord clamping
ECC: Early cord clamping
IRB: Institutional Review Board
MCH: Maternal and Child Health program
REC: Regional ethical committee
STA: Systematic text analysis
TBA: Traditional birth attendant
UCG: Uganda clinical guidelines
UDHS: Uganda district and health survey
UNICEF: United Nations International Children’s Emergency Fund
VHT: Village health team worker
WHO: World Health Organization
